# Supplementary material for: Early Re-Exploration versus Conservative Management for Postoperative Bleeding in Stable Patients after Coronary Artery Bypass Grafting: A Propensity Matched Study
Source: J Clin Med. 2023 May 7;12(9):3327. doi: 10.3390/jcm12093327 (PMC10179715; doi:10.3390/jcm12093327)
Supplement: Supplementary file 1 [file jcm-12-03327-s001.zip › jcm-2223493-supplementary.pdf]

Supplementary Table S1. Propensity score, logistic regression.

	Coefficient	Standard Error	P value	95% confidence interval	
Age	0.0108996	0.0081300	0.180	-0.0050349	0.0268341
Male sex	0.1353242	0.2153787	0.530	-0.2868102	0.5574586
Body surface area	0.3703407	0.2389886	0.121	-0.0980683	0.8387498
Diabetes					
diet	0.1500536	0.3382338	0.657	-0.5128725	0.8129797
oral	-0.1172776	0.2778239	0.673	-0.6618025	0.4272472
insulin	-0.0182513	0.3453902	0.958	-0.6952037	0.6587011
Smoking					
past	0.159199	0.1560541	0.308	-0.1466615	0.4650595
current	0.4253758	0.2475137	0.086	-0.0597422	0.9104937
Hypertension	0.1597344	0.1469703	0.277	-0.1283222	0.447791
Hypercholesterolemia	-0.1817113	0.1467164	0.216	-0.4692703	0.1058476
Preoperative dialysis	-1.197912	1.042455	0.251	-3.241086	0.8452624
Pulmonary disease	0.0879688	0.2414984	0.716	-0.3853593	0.5612968
Preoperative stroke					
transient	0.2357526	0.3127236	0.451	-0.3771743	0.8486795
stroke	-0.439563	0.628682	0.484	-1.671757	0.792631
Extracardiac arteriopathy	-0.2404877	0.2095109	0.251	-0.6511215	0.1701462
Number of diseased vessels					
2	0.2825012	0.4934924	0.567	-0.684726	1.249728
3	0.2215202	0.5053996	0.661	-0.7690449	1.212085
Left main disease	-0.0647119	0.1698154	0.703	-0.3975439	0.2681201
Left ventricular ejection fraction (LVEF), category					
fair	-0.0745296	0.1796082	0.678	-0.4265552	0.2774959
poor	-0.3687908	0.3746656	0.325	-1.103122	0.3655402
Number of distal anastomosis					
2	0.1788944	0.526024	0.734	-0.8520937	1.209882
3	-0.2580031	0.5481954	0.638	-1.332446	0.8164402
4	-0.1076718	0.5591905	0.847	-1.203665	0.9883214
5	-0.2355873	0.6384012	0.712	-1.486831	1.015656
On-pump surgery	-0.0810754	0.1878733	0.666	-0.4493002	0.2871495

Supplementary Table S2. Propensity score, diagnostic tests.

Variable	Unmatched (U) Matched (M)	Mean Treated Control	%bias	%reduct bias	t-test t-value / P-value
Age	U	65.789 / 64.978	8.3		1.23 / 0.219
	M	65.789 / 66.175	-3.9	52.3	-0.43 / 0.667
Male sex	U	0.86056 / 0.84964	3.1		0.45 / 0.655
	M	0.86056 / 0.86056	0.0	100.0	0.00 / 1.000
Body surface area	U	1.9664 / 1.9443	7.4		1.08 / 0.280
	M	1.9664 / 1.9708	-1.5	80.4	-0.15 / 0.880
Diabetes, diet	U	0.04781 / 0.03669	5.5		0.84 / 0.399
	M	0.04781 / 0.05578	-4.0	28.3	-0.40 / 0.688
Diabetes, oral	U	0.06773 / 0.07482	-2.8		-0.40 / 0.693
	M	0.06773 / 0.04382	9.3	-237.1	1.17 / 0.244
Diabetes, insulin	U	0.04382 / 0.04388	-0.0		-0.00 / 0.997
	M	0.04382 / 0.03187	5.8	1957.1	0.70 / 0.484
Smoking, past	U	0.52191 / 0.50072	4.2		0.62 / 0.537
	M	0.52191 / 0.53785	-3.2	24.8	-0.36 / 0.721
Smoking, current	U	0.11952 / 0.10000	6.2		0.94 / 0.349
	M	0.11952 / 0.09960	6.4	-2.0	0.71 / 0.476
Hypertension	U	0.59761 / 0.56259	7.1		1.03 / 0.303
	M	0.59761 / 0.61355	-3.2	54.5	-0.36 / 0.716
Hypercholesterolemia	U	0.50199 / 0.56331	-12.3		-1.80 / 0.072

	M	0.50199 / 0.50199	0.0	100.0	0.00 / 1.000
Preoperative dialysis	U	0.00398 / 0.01223	-9.2		-1.15 / 0.249
	M	0.00398 / 0.00797	-4.4	51.7	-0.58 / 0.563
Pulmonary disease	U	0.09562 / 0.08561	3.5		0.52 / 0.605
	M	0.09562 / 0.09163	1.4	60.2	0.15 / 0.879
Preoperative stroke, transient	U	0.05578 / 0.04676	4.1		0.61 / 0.539
	M	0.05578 / 0.06375	-3.6	11.6	-0.38 / 0.707
Preoperative stroke, stroke	U	0.01195 / 0.01655	-3.9		-0.54 / 0.592
	M	0.01195 / 0.01195	0.0	100.0	0.00 / 1.000
Extracardiac arteriopathy	U	0.13147 / 0.15252	-6.0		-0.86 / 0.390
	M	0.13147 / 0.14343	-3.4	43.2	-0.39 / 0.698
Number of diseased vessels, 2	U	0.22709 / 0.19065	9.0		1.34 / 0.181
	M	0.22709 / 0.23904	-2.9	67.2	-0.32 / 0.752
Number of diseased vessels, 3	U	0.73705 / 0.77122	-7.9		-1.18 / 0.240
	M	0.73705 / 0.7251	2.8	65.0	0.30 / 0.763
Left main disease	U	0.22709 / 0.23094	-0.9		-0.13 / 0.894
	M	0.22709 / 0.21514	2.8	-211.0	0.32 / 0.748
LVEF, category “fair”	U	0.19522 / 0.19784	-0.7		-0.10 / 0.924
	M	0.19522 / 0.20319	-2.0	-203.8	-0.22 / 0.824
LVEF, category “poor”	U	0.03586 / 0.04676	-5.5		-0.77 / 0.444
	M	0.03586 / 0.03586	0.0	100.0	0.00 / 1.000
Number of distal anastomosis, 2	U	0.24701 / 0.17842	16.8		2.56 / 0.011
	M	0.24701 / 0.25100	-1.0	94.2	-0.10 / 0.918

Number of distal anastomosis, 3	U	0.39841 / 0.45540	-11.5		-1.67 / 0.095
	M	0.39841 / 0.40637	-1.6	86.0	-0.18 / 0.856
Number of distal anastomosis, 4	U	0.27888 / 0.28417	-1.2		-0.17 / 0.864
	M	0.27888 / 0.24701	7.1	-502.7	0.81 / 0.418
Number of distal anastomosis, 5	U	0.04382 / 0.05180	-3.7		-0.53 / 0.596
	M	0.04382 / 0.05578	-5.6	-49.9	-0.61 / 0.539
On-pump surgery	U	0.81275 / 0.84101	-7.5		-1.11 / 0.265
	M	0.81275 / 0.84861	-9.5	-26.9	-1.07 / 0.285

**Sample**	**PsR2 / LRchi2**	**Mean bias**	**Med bias**	**Rubin's B**	**Rubin's R**
**Unmatched**	**0.019 / 25.99**	**6.1**	**5.5**	**35.2**	**0.97**
**Matched**	**0.011 / 7.37**	**3.5**	**3.2**	**24.2**	**0.89**
